# Pro-Inflammatory and Pro-Apoptotic Effects of the Non-Protein Amino Acid L-Azetidine-2-Carboxylic Acid in BV2 Microglial Cells

**DOI:** 10.3390/cimb44100308

**Published:** 2022-09-28

**Authors:** Jordan Allan Piper, Margo Iris Jansen, Sarah Thomas Broome, Kenneth J. Rodgers, Giuseppe Musumeci, Alessandro Castorina

**Affiliations:** 1 Laboratory of Cellular and Molecular Neuroscience (LCMN), School of Life Sciences, Faculty of Science, University of Technology Sydney, P.O. Box 123, Broadway, Sydney, NSW 2007, Australia; 2 Neurotoxin Research Group, School of Life Sciences, Faculty of Science, University of Technology Sydney, P.O. Box 123, Broadway, Sydney, NSW 2007, Australia; 3 Department of Biomedical and Biotechnological Sciences, Anatomy, Histology and Movement Science Section, School of Medicine, University of Catania, Via S. Sofia No. 87, 95123 Catania, Italy; 4 Research Center on Motor Activities (CRAM), University of Catania, Via S. Sofia No. 97, 95123 Catania, Italy

**Keywords:** L-azetidine-2-carboxylic acid, microglia, beets, multiple sclerosis, non-protein amino acid, neuroinflammation, environmental toxin

## Abstract

L-Azetidine-2-carboxylic acid (AZE) is a toxic non-protein coding amino acid (npAA) that is highly abundant in sugar and table beets. Due to its structural similarity with the amino acid L-proline, AZE can evade the editing process during protein assembly in eukaryotic cells and be misincorporated into L-proline-rich proteins, potentially causing protein misfolding and other detrimental effects to cells. In this study, we sought to determine if AZE treatment triggered pro-inflammatory and pro-apoptotic responses in BV2 microglial cells. BV2 microglial cells exposed to AZE at increasing concentrations (0–2000 µM) at 0, 3, 6, 12 and 24 h were assayed for cell viability (MTT) and nitric oxide release (Griess assay). Annexin V-FITC/propidium iodide (PI) staining was used to assess apoptosis. Real-time qPCR, Western blot and immunocytochemistry were used to interrogate relevant pro- and anti-inflammatory and other molecular targets of cell survival response. AZE (at concentrations > 1000 µM) significantly reduced cell viability, increased BAX/Bcl2 ratio and caused cell death. Results were mirrored by a robust increase in nitric oxide release, percentage of activated/polarised cells and expression of pro-inflammatory markers (*IL-1β*, *IL-6*, *NOS2*, *CD68* and *MHC-2a*). Additionally, we found that AZE induced the expression of the extracellular matrix degrading enzyme matrix metalloproteinase 9 (*MMP-9*) and brain derived neurotrophic factor (*BDNF*), two critical regulators of microglial motility and structural plasticity. Collectively, these data indicate that AZE-induced toxicity is associated with increased pro-inflammatory activity and reduced survival in BV2 microglia. This evidence may prompt for an increased monitoring of AZE consumption by humans.

## 1. Introduction

In nature, in addition to the 20 canonical amino acids involved in protein assembly in eukaryotic cells, there are hundreds of plant-derived amino acids, namely non-protein amino acids (npAAs) [1]. In some cases a npAA can mimic a protein amino acid and replace it in a physiological process; including as a substrate in protein synthesis [1]. Some of these ‘proteinogenic’ npAAs are secreted by plants as deterrents against predation, as well as growth inhibitors for competing plants in a phenomenon known as allelopathy [2]. In part owing to their relatively low concentrations and their negligible nutritional value, npAAs have been often overlooked, although there is growing evidence suggesting that prolonged exposure or their undetected entry in the food chain may cause significant biochemical changes and pose a risk to human health [3,4].

L-Azetidine-2-carboxylic acid (AZE), firstly identified by Dr Rubenstein in table beets and sugar beets in 2006 [5], is a proteinogenic npAA potentially implicated in multiple sclerosis (MS) pathogenesis [6]. AZE shares high structural similarity with L-proline, a protein-coding amino acid that is abundant in collagen, keratin, hemoglobin and core myelin proteins [5]. Due to its ability to evade recognition by transfer RNAs, the impostor AZE evades the editing process and is erroneously misplaced in lieu of the authentic L-proline, causing structural changes to L-proline-containing proteins [6]. According to Rubenstein’s initial theory [6], more recently supported by an interesting work from his colleagues [7], AZE misplacement increases the immunogenicity of certain myelin proteins, to likely initiate the autoimmune events leading to oligodendrogliopathy [8] and microgliosis [9], two known pathogenic features of MS.

In humans, myelination of the central nervous system (CNS) occurs during late gestational age and perinatal period, to then progressively reduce during early childhood development [10]. It is during this developmental stage that myelin, myelin-producing cells and other CNS cell types are more plastic, but also more susceptible to pathological changes [11]. In view of AZE ability to misincorporate into proteins and cause structural alterations, it is reasonable to hypothesise that the detrimental effects of AZE can extend beyond myelin or oligodendrocytes, and perhaps affect more broadly other cell populations within the CNS, including microglia.

Perturbations in protein assembly can lead to protein misfolding [12], and the accumulation of misfolded proteins triggers endoplasmic reticulum (ER)-stress [13]. However, the ER is equipped with highly specific signalling pathways called the unfolded protein response (UPR) to cope with the accumulation of unfolded or misfolded proteins [12].

Recently, UPR has been studied in myeloid cells, where it was demonstrated to act as a fundamental proteostatic pathway to coordinate inflammatory responses [14]. Microglia, the resident innate immune cell of the CNS, are responsible for the ongoing CNS surveillance, the release of pro-inflammatory factors and act as scavengers to clear cellular and toxic debris [15]. Depending on the nature of the insult/stimulus, microglial cells undergo dynamic morphological and functional changes ranging from quiescent/resting state to either polarised/activated pro-inflammatory or anti-inflammatory states [16]. Current evidence suggests that prolonged microglial polarisation may promote neuronal damage and aggravate oligodendroglial pathology, similar to what we see in neuroinflammatory diseases such as MS [17,18]. For such reasons, aberrantly activated microglia have also been implicated in the pathogenic cascade of a diverse range of neurodegenerative diseases, including demyelinating ones [18].

In the recent work by Sobel et al., [7], the authors provide evidence that AZE supplementation in rodents triggers the UPR in the CNS white matter, which is associated with the appearance of microglial nodules and the activation of pro-inflammatory signalling. However, whether this is due to a direct effect on microglia or it is secondary to oligodendrocyte or myelin damage remains to be established. To address this issue, using an in vitro model of AZE toxicity, we aimed at investigating the biological effects of AZE exposure in murine BV2 microglial cells.

## 2. Materials and Methods

### 2.1. Cell Culture and Treatments

The study was carried out using the murine microglial BV2 cell line. Cells were grown in Dulbecco’s modified Eagle’s medium (DMEM)/F12 and were supplemented with 10% heat-inactivated fetal bovine serum (FBS), 2 mM L-glutamine, 100 U/mL penicillin and 200 μg/mL streptomycin (Sigma–Aldrich, Castle Hill, NSW, Australia). Cells were seeded in T25 flasks at a density of 1 × 10^5^ cells. Cells were maintained at 37 °C in a humidified atmosphere with 5% CO_2_ in air. Cells were incubated until they reached 75–80% confluence before being used for experimental testing. Upon treatment, cell media was replaced with either fresh DMEM (for untreated controls), or media containing increasing concentrations of AZE (0–2000 µM; A0760, Sigma-Aldrich, Castle Hill, NSW, Australia). For this purpose, a 100 mM AZE stock solution was freshly prepared and diluted as required. Cells exposed or not to AZE were then placed in a CO_2_ incubator for different time points, depending on the assay.

### 2.2. MTT Assay

To assess cell viability, we used the Cell Proliferation Kit I (MTT) following manufacturer’s instructions (Cat #11465007001, Sigma-Aldrich, Castle Hill, NSW, Australia). Cells were seeded into 96-well plates at a concentration of 1 × 10^4^ cells/well. DMEM containing 0.5 mg/mL 3-[4,5-dimethylthiazol-2-yl]-2,5-diphenyltetrazolium bromide (MTT) (Sigma–Aldrich) was added in each well. Following incubation for 4 h at 37 °C, medium was removed, and 100 μL of DMSO was added. Formazan formed by the cleavage of the yellow tetrazolium salt MTT was analysed using a spectrophotometer by measuring absorbance change at 550–600 nm using a microplate reader.

### 2.3. Griess Assay

Griess assay was performed as indicated in previous work [19]. The assay was specifically used to measure the relative abundance of nitric oxide (NO) released by BV2 microglia upon stimulation with AZE. Cells were seeded at 2 × 10^4^ cells per well in a 96-well plate and incubated at 37 °C with 5% CO_2_ until cells reached 80% confluence. Cells were treated with either control media or increasing concentrations of AZE (0, 125, 250, 500, 1000 and 2000 µM) for 0, 3, 6, 12 and 24 h. The supernatant was collected and placed into a new 96-well plate. A total of 100 µL of freshly prepared Griess reagent was then added to each well and incubated at room temperature for 15 min on a slow oscillation protected from light. Absorbance was measured at 540 nm using the TECAN infinite M1000-PRO ELISA reader. Optical density values from each group were recorded and reported as a percentage of control.

### 2.4. RNA Extraction and cDNA Synthesis

Total RNA was extracted from untreated BV2 cells (Ctrl) or cells exposed to 1000 µM AZE for 6 and 24 h, respectively. Briefly, cells were lysed using 1 mL TRI reagent (Sigma-Aldrich, Castle Hill, NSW, Australia) and 0.2 mL chloroform and precipitated with 0.5 mL 2-propanol (Sigma-Aldrich) [20]. Pellets were washed twice with 75% ethanol and air-dried. RNA concentrations were calculated using NanoDrop™ 2000 (ThermoFisher Scientific, Waltham, MA, USA). A total of 1 µg of total RNA were loaded in each cDNA synthesis reaction. cDNA synthesis was conducted using the T1000 thermal cycler (Bio-Rad, Gladesville, NSW, Australia) in a final volume of 20 µL. Each reaction contained 1 μg of RNA diluted in a volume of 11 µL, to which we added 9 µL of cDNA synthesis mix (Tetro cDNA synthesis kit; Bioline, Redfern, NSW, Australia). Samples were incubated at 45 °C for 40 min followed by 85 °C for 5 min. Finally, cDNA samples were diluted at a final concentration of 10 ng/mL in milliQ H_2_O and stored at −20 °C until use.

### 2.5. Real Time Quantitative PCR Analysis

Real-time qPCR analyses were carried out as previously reported [20,21], with minor modifications. For each gene of interest, qPCRs were performed in a final volume of 10 μL, which comprised 3 μL cDNA, 0.4 μL milliQ H_2_O, 5 μL of iTaq Universal SYBR green master mix (BioRad, Gladesville, NSW, Australia) and 0.8 μL of the corresponding forward and reverse primers (5 μM, Sigma-Aldrich, Castle Hill, NSW, Australia) to obtain a final primer concentration of 400 nM. The primers are described in Table 1. Reaction mixtures were loaded in Hard-Shell^®^ 96-Well PCR Plates, and four genes of interest were tested in each run using the CFX96 Touch™ Real-Time PCR Detection System (Bio-Rad, Gladesville, NSW, Australia). Instrument settings were as follows: (1) 95 °C for 2 min, (2) 60 °C for 10 s, (3) 72 °C for 10 s, (4) plate read, (5) repeat step 2 to 4, for 45 cycles. For the melting curve analyses, settings were (1) 65 °C for 35 s, (2) plate read, (3) repeat step 1–2 for 60 times). To examine changes in expression, we analysed the mean fold change values of each sample, calculated using the ΔΔCt method, as previously described by Schmittgen and Livak [22]. PCR product specificity was evaluated by melting curve analysis, with each gene showing a single peak (data not shown).

### 2.6. Protein Extraction and Western Blot Analyses

Proteins from either untreated (Ctrl) or 1000 µM AZE-treated BV2 microglial cells at 6 and 24 h were extracted using radioimmunoprecipitation assay (RIPA) buffer (Sigma-Aldrich, Castle Hill, NSW, Australia) containing 1× Protease Inhibitor cocktail (cOmplete™, Mini, EDTA-free Protease Inhibitor Cocktail, Sigma-Aldrich, Castle Hill, NSW, Australia). Protein was quantified using the Bicinchoninic-Acid (BCA) Assay Protein Assay Kit (ThermoFisher Scientific, North Ryde, NSW, Australia) according to manufacturer’s protocol and measured using the TECAN infinite M1000-PRO ELISA plate reader at 562 nm adsorbance.

Sample lysates were prepared by adding 3.75 μL of Laemmli Buffer (Bio-Rad, Gladesville, NSW, Australia) containing β-mercaptoethanol (Sigma-Aldrich, Castle Hill, NSW, Australia) mixture, (ratio 1:9 *v*/*v*) to 30 μg protein in a final volume of 15 μL. Samples were then heated for 10 min at 70 °C to denature proteins [23]. Proteins were then separated by SDS-polyacrylamide gel electrophoresis (SDS-PAGE) using 4–20% mini gels (Bio-Rad, Criterion 15-well Mini-Protean SFX), alongside with 5 µL of the molecular weight ladder/marker (BioRad Pre-stained HyperLadder Precision Plus Protein™; BioRad, Gladesville, NSW, Australia). Gels were transferred to a polyvinylidene fluoride (PVDF) membrane using the Trans-Blot Turbo instrument (BioRad). Once terminated, membranes were immediately placed in a container filled with TBS/0.1% Tween-20 (Sigma-Aldrich, Castle Hill, NSW, Australia) (TBST 1×) to wash out any residues during transfer. To block non-specific binding sites, membranes were blocked for 1 h in 5% dry non-fat skim milk in TBST with slow agitation (50–60 rpm).

Membranes were incubated with appropriately diluted primary antibodies in blocking buffer overnight at 4 °C with slow agitation. Primary antibodies used in this study and related dilutions are shown in Table 2. This was followed by incubation with host-specific secondary antibodies. Membranes were then placed in a container with 1 × TBST and washed rapidly three times, followed by three further 5 min washes. Finally, membranes were incubated in secondary antibody (HRP-conjugated goat anti-rabbit IgG; BioRad) for 1 h at room temperature, diluted in blocking buffer. The membranes were then washed once again as previously described to remove excess secondary antibody. Imaging was then performed on the Bio-Rad ChemiDoc MP Imaging System (BioRad). To detect bands, we utilized Clarity Western ECL Blotting Substrate (BioRad). Densitometric analyses of bands were computed using NIH ImageJ (https://imagej.nih.gov/ij/download/ (accessed on 14 October 2021)). Optical densities of target proteins were normalised to those of loading controls (GAPDH).

### 2.7. Flow Cytometry

Apoptosis and necrosis were detected by differential staining with annexin V (early and late apoptotic cells) and propidium iodide (PI) (necrotic cells only) using the Dead Cell Apoptosis Kit with Annexin V Alexa Fluor 488 and PI (#V13241; ThermoFisher Scientific), according to manufacturer’s instructions. Briefly, BV2 cells were treated with 1000 µM of AZE for 24 h. Cells were washed in cold PBS, re-centrifuged and 1 × 10^6^ cells were suspended in 1 × annexin-binding buffer. Cells were stained with Alexa Fluor 488 Annexin-V and propidium iodide (PI) for 15 min then analysed by flow cytometry. Unstained and single stained controls were used for compensation to correct for fluorescence photobleaching and gating (Supplementary Figure S1). FloJo software was used to analyse and curate flow cytometry data.

### 2.8. Immunocytochemistry

Sterile tissue culture coverslips (22 mm Ø, Sarstedt, SA, Australia) were coated with poly-L-lysine (100 µg/mL in sterile milliQ H_2_O) (Sigma-Aldrich, Castle Hill, NSW, Australia) prior to cell culturing. Cells were cultured on coverslips at 1 × 10^4^ cells in normal growth media or supplemented with AZE (1000 µM) for 6 or 24 h. Cells were then fixed with 4% filtered paraformaldehyde (PFA: 4% in PBS pH 7.4) (Sigma-Aldrich, Castle Hill, NSW, Australia) for 15 min at room temperature. Coverslips were then washed three times and permeabilised in PBS containing 0.25% Triton X-100 (Sigma-Aldrich, Castle Hill, NSW, Australia), followed 3× washes in PBS for 5 min [24]. Non-specific binding of antibodies was prevented by incubating coverslips with 1% BSA in PBST for 30 min. Once completed, the cells were incubated in diluted primary antibody (using 1% BSA in PBST) in a humidified chamber overnight at 4 °C (please see Table 2 for dilutions). The next day, the primary antibody was removed with 3× washes in PBS for 5 min. Cells were then incubated in the dark, with fluorophore-conjugated secondary antibodies (Alexa Fluor-488 or -594 anti-rabbit IgG) in 1% BSA in PBST overnight at 4 °C with gentle oscillation. Secondary antibody solution was then removed, and cells were washed again three times with PBS for 5 min in the dark. Cell nuclei were counterstained with 0.3 µg/mL DAPI for 1 min (DNA stain) (Cell Signalling Biotechnologies, Danvers, MA, USA) followed by a quick (1 min) rinsed with PBS. Finally, coverslips were mounted using a drop of mounting medium (Prolong Antifade Gold, Cell Signalling Biotechnologies) and sealed using nail polish to be stored in the dark at −20 °C before imaging.

### 2.9. Statistical Analysis

Statistical analyses were performed using GraphPad Prism software v9.3 (GraphPad Software, La Jolla, CA, USA). Pairwise comparisons were analysed by Student *t*-test. Comparisons between three or more groups were analysed by One-Way ANOVA followed by Sidak or Dunnett’s post hoc test, as appropriate. *p*-values less than 0.05 were considered statistically significant.

## 3. Results

### 3.1. Dose–Response and Time Course Study of the Effects of AZE on Cell Morphology, Viability and Nitric Oxide (NO) Release

**Figure 1:**
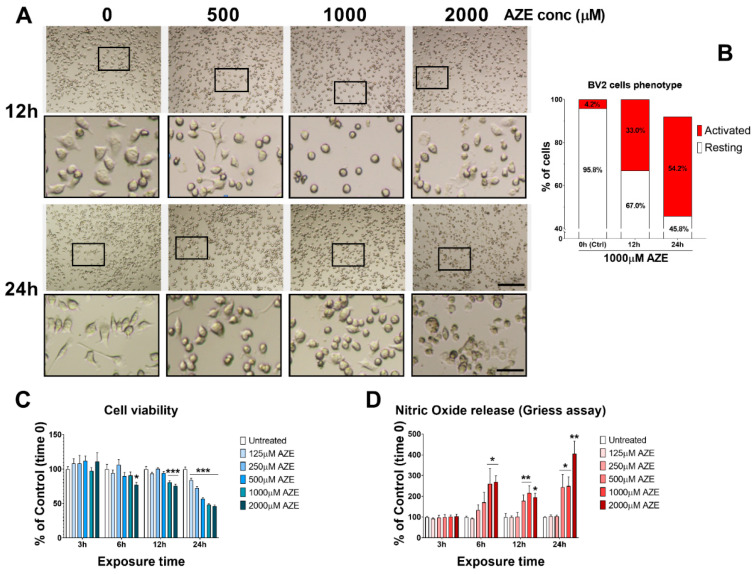
Dose–response effects of AZE exposure on BV2 microglial cell viability and inflammatory response. (**A**) Morphological changes seen in microglial BV2 cells following various AZE concentrations (0, 500, 1000, 2000 μM) after 12 and 24 h. Representative photomicrographs were taken using a bright field microscope with the embossing filter settings. Magnification = 10×, scale bar = 200 μm. Black squares indicate the ROI shown at higher magnification in insets. Insets below each photomicrograph show high magnification details of cellular morphology. Magnification = 40×, scale bar = 50 μm. (**B**) Phenotypic presentation of BV2 cells (Activated vs. Resting). % of cells of each phenotype was determined by counting the # of cells that showed signs of activation (flat and swollen) vs. the total number of cells per region of interest (ROI) and expressed as a percentage (n = 5 ROI × 3 batches of cells). (**C**) Cell viability, measured by MTT assay. Cells were treated with varying AZE concentrations (0, 125, 250, 500, 1000, 2000 µM) for 3, 6, 12 or 24 h. * *p* < 0.05 or *** *p* < 0.001, as determined by ANOVA followed by Dunnett’s post hoc test. (**D**) Nitric oxide release, assessed using the Griess assay. Cells were treated as in C and NO levels were measured in culture media. Values are reported as the percentage NO release of untreated controls. Data reported as mean ± SEM. * *p* < 0.05, ** *p* < 0.01, as determined by ANOVA followed by Dunnett’s post hoc test.

To determine if AZE exposure triggered gross phenotypic changes to BV2 microglia, we exposed cells to increasing concentrations of AZE (0, 500, 1000 and 2000 µM) at two different time intervals (12 and 24 h, respectively) and assessed cell morphology on a bright field microscope, using the embossing filter setting. The latter setting allows to discriminate microglial cells that acquire a flattened morphology (typical of activated / polarised BV2 cells). As shown in Figure 1A, BV2 cells exposed to increasing AZE concentration undergo considerable morphological changes in both, size, shape and overall appearance of cell somata and processes. Gross stereological assessment of cells displaying features of resting (small/raised somata) or polarised state (swollen/enlarged flat somata with rectracted processes) was performed in cells that were exposed to 1000 µM AZE for 12 and 24 h (Figure 1B). At time 0, only a small percentage of cells showed activated-like morphology (4.2% activated vs. 95.8% resting). After 12 h, there was already a remarkable increase in the percentage of activated microglia (33% activated vs. 67% resting), which was further increased after 24 h (54.2% activated vs. 45.8%).

Cell viability assessment using the MTT assay revealed a higher than expected reduction in nicotinamide adenine dinucleotide phosphate (NADPH)-dependent conversion of the yellow tetrazolium salt (3-(4,5-dimethylthiazol-2-yl)-2,5-diphenyltetrazolium bromide or MTT) to purple formazan crystals (Figure 1C), suggesting robust detrimental effects of AZE on BV2 cell metabolism. Time-course assessments demonstrated significant reduction in viability only at 2000 µM AZE (* *p* < 0.05 vs. Ctrl) after 6 h, whereas both 1000 and 2000 µM AZE significantly reduced viability at 12 h (*** *p* < 0.001 vs. Ctrl). Lastly, AZE concentrations from 125–2000 µM reduced viability after 24 h exposure to the npAA (*** *p* < 0.001 vs. Ctrl).

To examine if AZE treatment also influenced the release of nitric oxide (NO) in the supernatant (indicative of pro-inflammatory activity) we used the Griess reagent assay. Analyses of relative NO levels (shown as % of Ctrl) indicated that AZE, at the highest concentrations tested, significantly increased NO levels both at 6, 12 and 24 h (Figure 1D). Specifically, at 6 h, both 1000 and 2000 µM AZE reliably increase NO production (* *p* < 0.05 vs. Ctrl), whereas after 12 h, both 500, 1000 and 2000 µM AZE produced similar effects (** *p* < 0.01 vs. Ctrl at 500 and 1000 µM and * *p* < 0.05 vs. Ctrl at 2000 µM, respectively). A more robust increase in NO levels was seen after 24 h, with AZE significantly increasing NO at 500 and 1000 µM (* *p* < 0.05 vs. Ctrl) and 2000 µM (** *p* < 0.01 vs. Ctrl).

### 3.2. Effects of AZE Exposure on Bcl2 and BAX Protein Expression in Murine BV2 Microglial Cells

**Figure 2:**
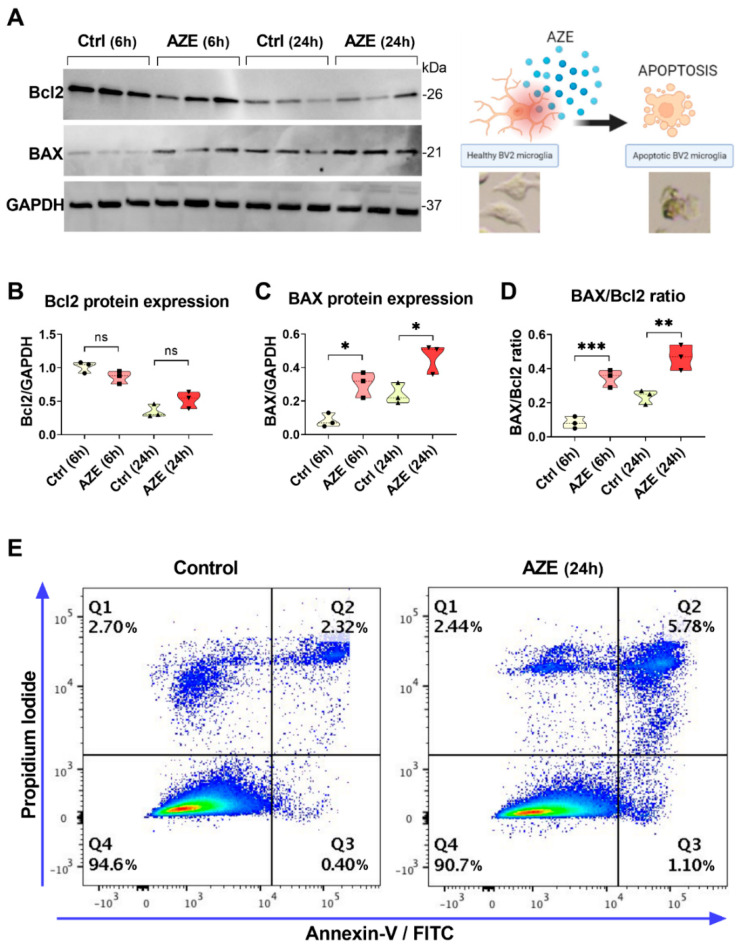
(**A**) Representative Western blots showing Bcl2 and BAX protein expression in BV2 cells cultured in the presence or not of AZE (1000 µM) for 6 or 24 h. GAPDH was used as loading control. (**B**–**D**) Violin plots depicting results from densitometric analyses of Bcl2 and BAX blots as well as BAX/Bcl2 ratios. Results shown are the mean ± SEM of two independent determinations, each run in triplicate. Ns = not significant. * *p* < 0.05, ** *p* < 0.01 or *** *p* < 0.001 vs. Ctrl at the corresponding time point, as determined by ANOVA and Sidak’s post hoc test. (**E**) The incidence of apoptotic cells was examined by flow cytometry using Annexin V-FITC/PI staining. The experiment was repeated twice with overlapping results.

To investigate if AZE-driven reduction in cell viability was, at least in part, due to apoptosis, we determined the expression levels of the anti-apoptotic Bcl2 and pro-apoptotic BAX proteins by Western blot analyses. As indicated in Figure 2A, cells were either left untreated or exposed to 1000 µM AZE for 6 or 24 h.

Bcl2 protein expression was not significantly affected by AZE treatment at any of time points tested (*p* > 0.05 vs. Ctrl; Figure 2B). In contrast, levels of the pro-apoptotic BAX protein were significantly increased both at 6 and 24 h (* *p* < 0.05 vs. Ctrl at the corresponding times; Figure 2C). We further analysed BAX/Bcl2 ratio, an indicator of apoptosis susceptibility in BV2 cells exposed to the same experimental conditions. We found that, already after 6 h after AZE exposure, the ratio was significantly increased (*** *p* < 0.001 vs. Ctrl (6 h)) and was still augmented at 24 h (** *p* < 0.01 vs. Ctrl (24 h); Figure 2D).

Finally, we conducted Annexin V-FITC/PI staining on BV2 cells exposed or not to 1000 µM AZE for 24 h. The experiment demonstrated a remarkable increase in the percentage of early apoptotic and necrotic or late apoptotic cells (about 5.8%) compared to the control group (2.3%) (Figure 2E).

### 3.3. Effects of AZE Exposure on the mRNA Expression of Pro- and Anti-Inflammatory Markers in Murine BV2 Microglial Cells

To explore whether AZE treatment alters the inflammatory profile of BV2 cells, we analysed the expression of a panel of pro- and anti-inflammatory markers by real-time quantitative polymerase chain reaction (qPCR).

**Figure 3:**
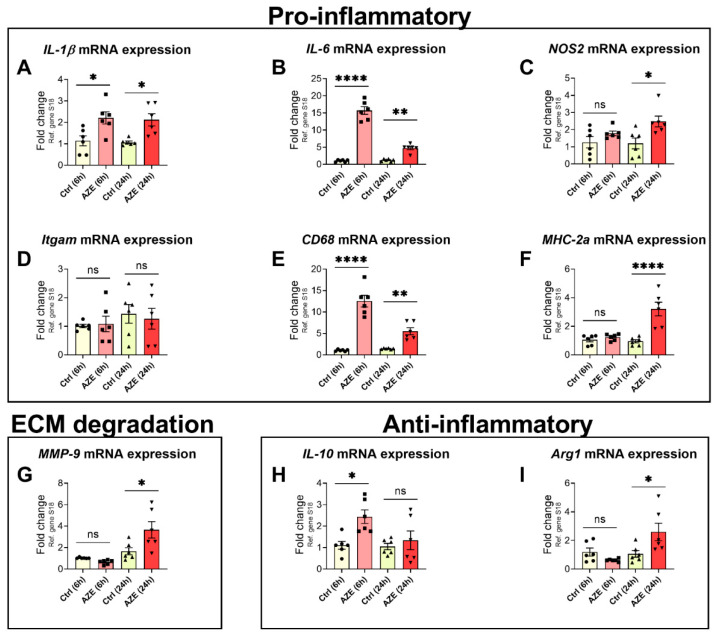
Gene expression of a panel of pro- and anti-inflammatory genes in BV2 microglial cells after 1000 µM AZE treatment for 6 and 24 h. Relative fold-changes were calculated using the ΔCt method after normalization to the S18 housekeeping gene. Box-and-whisker plots depict the differential expression of pro-inflammatory (**A**–**F**) *IL-1β, IL-6, NOS2, Itgam, CD68* and *MHC-2a*, (**G**) extracellular matrix (ECM) degradation *(MMP-9)* and (**H**–**I**) anti-inflammatory *IL-10* and *Arg1* transcripts. Results are presented as mean fold changes with respect to no treatment (Ctrl) ± SEM. Data represents n = 6 samples per group. * *p* < 0.05, ** *p* < 0.01 or **** *p* < 0.0001; as determined by ANOVA followed by Sidak’s post hoc test. Ns = not significant.

As depicted in Figure 3A, AZE triggered a significant increase in interleukin-1β (*IL-1β*) gene expression both after 6 and 24 h (* *p* < 0.05 vs. Ctrl). In contrast, interleukin-6 (*IL-6*) gene induction was more prominent at 6 h (>15-fold of Ctrl, **** *p* < 0.0001 vs. Ctrl) vs. 24 h treatment (** *p* < 0.01 vs. Ctrl) (Figure 3B). *NOS2*, the gene encoding for inducible nitric oxide synthase (iNOS)—the enzyme that catalyses the production of NO—was only marginally increased after 6 h (*p* > 0.05) but was significant at 24 h (* *p* < 0.05; Figure 3C). The expression level of *Itgam*, the gene encoding for the macrophage marker CD11b, was not elevated in response to AZE (*p* > 0.05; Figure 3D). However, CD68 (aka microsialin)—another myeloid cell marker, was robustly induced after 6 h AZE exposure (>12-fold of Ctrl, **** *p* < 0.0001) and remained elevated at 24 h (** *p* < 0.01) (Figure 3E). Analyses of the expression of the major histocompatibility complex IIa (MHC-2a), which serves a critical role in the induction of immune responses through presentation of antigenic peptides to lymphocytes, was not induced at 6 h post-AZE treatment, but significantly up-regulated at 24 h (**** *p* < 0.0001; Figure 3F).

Expression of metalloproteinase-9 (MMP-9), an extracellular matrix degrading enzyme [25], was also robustly up-regulated by AZE treatment, but only at 24 h (* *p* < 0.05; Figure 3G).

Finally, we also interrogated two anti-inflammatory genes: interleukin-10 (IL-10) and arginase 1 (Arg1) [26]. Real-time qPCR revealed a significant up-regulation of the former at 6 h (* *p* < 0.05) but not at 24 h (*p* > 0.05) (Figure 3H), whereas the latter was significantly up-regulated only after 24 h (* *p* < 0.05).

### 3.4. Effects of AZE Exposure on IL-6 and Arg1 Protein Expression in Murine BV2 Microglial Cells

To confirm if the effects of AZE treatment on pro- and anti-inflammatory genes could also be appreciated at the protein level, we measured the protein expression of both IL-6 (pro-inflammatory) [27] and Arg1 (anti-inflammatory) [27] by Western blot.

**Figure 4:**
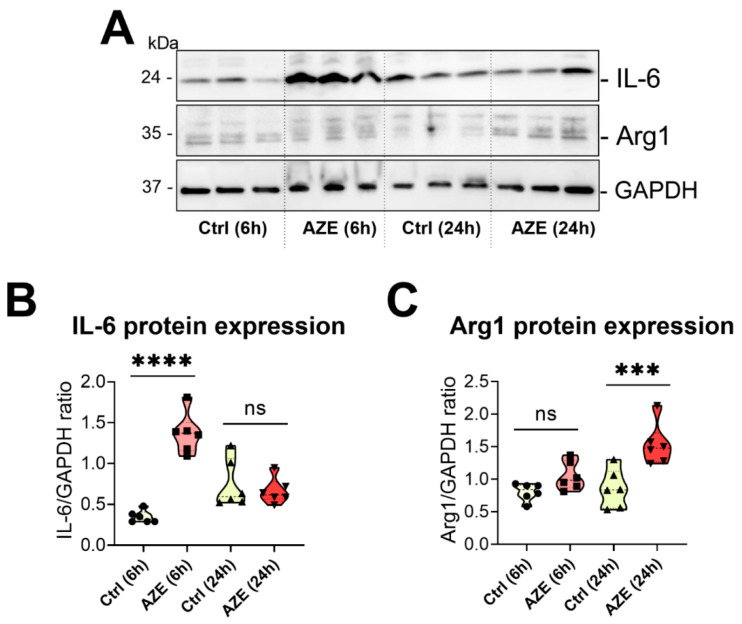
(**A**) Representative Western blots showing the protein expression of IL-6 and Arg1 in BV2 cells cultured or not in the presence of AZE (1000 µM) for 6 or 24 h. (**B**,**C**) Violin plots of bands densities demonstrating the effects of AZE on the expression of IL-6 and Arg1 at the indicated times. Data reported is the mean ± SEM, from two independent experiments using separate batches of cells (n = 6). GAPDH was used as loading control. Ns = not significant. *** *p* < 0.001 or **** *p* < 0.0001 vs. Ctrl at the indicated time, as determined by ANOVA followed by Sidak’s post hoc test.

In BV2 cells treated with AZE, IL-6 protein expression was heavily induced at 6 h (**** *p* < 0.0001 vs. Ctrl (6 h)); however, expression returned to baseline after 24 h (*p* > 0.05) (Figure 4A,B). In contrast, Arg1 expression was not affected after 6 h AZE (*p* > 0.05) but was remarkably increased at the 24 h time point (*** *p* < 0.001 vs. Ctrl) (Figure 4A,C).

### 3.5. Effects of AZE Treatment on the Gene and Protein Expression of the Pro-Inflammatory Marker Allograft Inflammatory Factor 1 (AIF1)/Ionized Calcium-Binding Adapter Molecule 1 (Iba1)

To better characterise AZE pro-inflammatory activities in BV2 microglia, cells were exposed to the same concentration of the npAA (1000 μM) for 6 or 24 h and the expression of *AIF1* (gene) and its protein product (Iba1) were interrogated using different experimental means.

**Figure 5:**
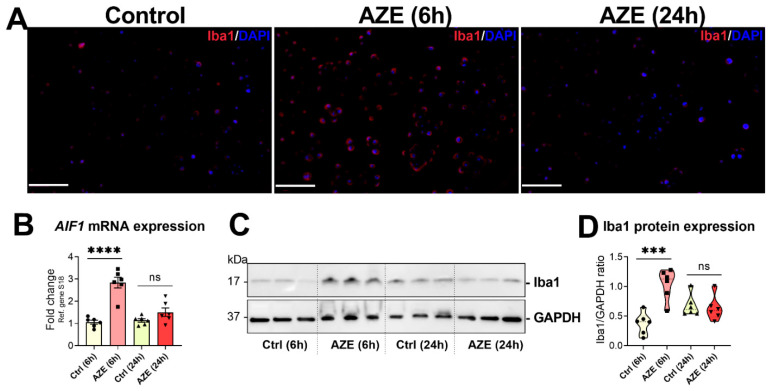
AZE treatment induces *AIF1*/Iba1 expression in BV2 cells. (**A**) Representative photomicrographs of Iba1+ staining in the BV2 microglial cell line. Cells were either left untreated or treated with AZE as indicated and then processed for immunocytochemistry (ICC) as detailed in Section 2. Red = Iba1+ cells (Alexa Fluor 594). DAPI = nuclei. Scale bar = 50 µm. (**B**) *AIF1* gene expression, as determined by real-time qPCR. Fold changes were calculated using the ΔCt method, with baseline expression set to 1. Data is the mean ± SEM (n = 6). **** *p* < 0.0001 vs. Ctrl at the indicated time points. (**C**,**D**) Western blot analyses of Iba protein expression and densitometric analyses of blots. Data reported is the mean ± SEM, from two independent experiments using separate batches of cells (n = 6). GAPDH was used as loading control. Ns = not significant. *** *p* < 0.001 or **** *p* < 0.0001 vs. Ctrl at the indicated time, as determined by ANOVA followed by Sidak’s post hoc test.

Immunofluorescence revealed a remarkable enhancement in Iba1+ signal in BV2 cells exposed to AZE for 6 h; however, signal intensity returned to normal level by 24 h (Figure 5A). Similarly, *AIF1* transcripts were up-regulated after 6 h AZE (**** *p* < 0.0001 vs. Ctrl (6 h)), but almost returned to untreated levels within 24 h (Figure 5B). These results were corroborated by Western blots, showing significantly increased Iba1 protein levels at 6 h (*** *p* < 0.001), but not at 24 h (*p* > 0.05) (Figure 5C,D).

### 3.6. Effects of AZE Treatment on the Gene and Protein Expression of Brain-Derived Neurotrophic Factor (BDNF)

BDNF is a neurotrophic factor known to play an important role in microglia inflammatory responses [28]. Here, we assessed BDNF mRNA and protein expression in cells exposed to 1000 μM AZE for 6 and 24 h.

**Figure 6:**
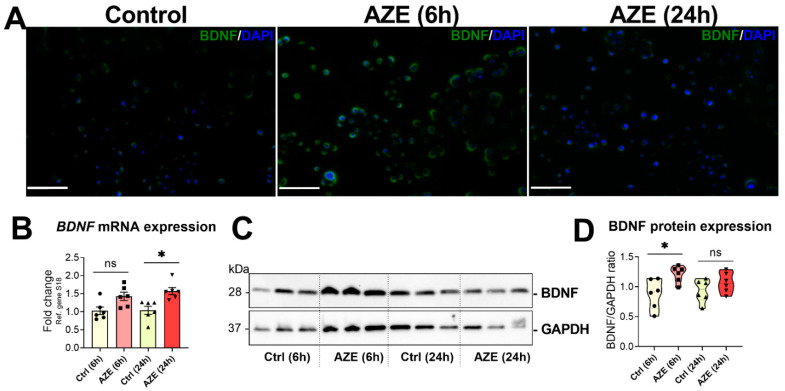
AZE treatment induces BDNF expression in BV2 cells. (**A**). Representative photomicrographs of BDNF+ staining in the BV2 microglial cell line. Cells were either left untreated or treated with AZE as indicated and then processed for immunocytochemistry (ICC) as detailed in Section 2. Red = BDNF+ cells (Alexa Fluor 488). DAPI = nuclei. Scale bar = 50 µm. (**B**). *BDNF* transcript levels, as determined by real-time qPCR. Fold changes were calculated using the ΔCt method. Data is the mean ± SEM (n = 6). * *p* < 0.05 vs. Ctrl at the indicated time points. (**C**,**D**) Western blot analyses of Iba protein expression and densitometric analyses of blots. Data reported is the mean ± SEM, from two independent experiments using separate batches of cells (n = 6). GAPDH was used as loading control. Ns = not significant. * *p* < 0.05 vs. Ctrl at the indicated time, as determined by ANOVA followed by Sidak’s post hoc test.

ICC demonstrated a rapid increase in BDNF+ staining in cells after 6 h AZE (Figure 6A), which was attenuated at 24 h. Interestingly, BDNF mRNA expression was slightly (but not significantly) increased at 6 h post-AZE treatment (*p* > 0.05 vs. Ctrl (6 h)); however, the increase was significant at 24 h (* *p* < 0.05 vs. Ctrl (24 h)) (Figure 6B). At the protein level, BDNF expression was significantly increased at 6 h AZE (* *p* < 0.05), whereas the increased was no longer significant at 24 h (Figure 6C,D).

## 4. Discussion

To the best of our knowledge, this is the first study portraying the pro-inflammatory and pro-apoptotic effects of acute AZE exposure in a microglial cell line.

Microglial cells are involved in several brain functions, and are recognised as “agents of the CNS” [29]. Their activities span from the clearing of cell debris after injury or pathogen attack (the well-known cell scavenging function) [30], going through the control of inflammatory responses [31], to latest reports highlighting microglial role in myelination [32]. Environmental factors able to disrupt the functionality of these cells may have a strong negative impact on CNS homeostasis, contributing to the onset of neurodegenerative diseases, including MS [33].

There is a growing body of work suggesting that certain npAAs produced by plants have the clear potential to adversely affect human health [1,34]. The exact pathogenic mechanisms are yet to be revealed; however, several theories have pinpointed the existence of geographical and historical links with the increased prevalence of CNS disorders among the population exposed to these toxins over a prolonged period of time [6,35,36], instigating researchers to conduct further investigations on this class of environmental risk factors.

Due to its similarity to L-proline, AZE is capable of entering cells, being charged onto the tRNA^Pro^, where it can evade editing by aminoacyl-tRNA synthetase and be misincorporated into proline-containing proteins during protein assembly [37]. Since this mistaken incorporation is thought to be a random process dependent on the relative concentrations of AZE and proline, proline-rich proteins are more likely to contain AZE. Misincorporation of AZE is thought to alter the structural conformation of newly assembled proteins, resulting in protein misfolding and, consequently, ER-stress [38]. Sobel and collaborators have recently shown that administration of AZE to laboratory animals (especially young mice) triggers oligodendrocytes swelling, formation of microglial nodules and cell polarization [7], and providing essential proof-of-concept data to indicate that AZE consumption is associated with some degree of CNS pathogenicity. It is unclear though if AZE-induced protein misfolding is restricted to oligodendrocytes or it extends more globally to different CNS cell types, including microglia. In fact, whereas protein misfolding in oligodendrocytes could potentially activate neighbouring microglia—causing cell activation—it cannot be excluded that AZE may directly trigger an inflammatory response after it enters the cell. This idea is supported by emerging evidence providing a link between ER stress-mediated activation of the UPR in microglia and cell polarization [14] and previous evidence of cell death and mitochondrial dysfunction in SH-SY5Y neuronal-like cells [39].

In this study, using the BV2 microglial cell line, which shares several biochemical and transcriptional features with primary microglia [40,41], we aimed at determining if AZE in the culture media would trigger cell polarization and other detrimental effects. Our findings revealed that a relatively brief exposure to supraphysiological concentrations of AZE was capable of triggering a barrage of pro-inflammatory signals consistent with overt M1-like polarization. Cells acquired the typical morphology of activated microglia, with flattened and swollen somata and expressed high levels of pro-inflammatory mediators (*IL-1β*, *IL-6*, *NOS2*), the neurotrophic factor BDNF and other myeloid cell activation markers (*CD68, MHC-2a*, *MMP-9* and *AIF1*). In parallel, expression of the anti-inflammatory markers *IL-10* and *Arg1* were also increased. This an expected occurrence, likely due to the physiological attempt to regain homeostatic control over the inflammatory response by a subset of cells.

Unexpectedly, expression of *Itgam*—the gene encoding for the myeloid marker CD11b—was not affected by AZE. This is particularly interesting as CD11b induction occurs via a NO-dependent mechanism [42], and AZE reliably increased NO release as well as the expression of the *NOS2* gene. However, it should be noted that gene expression data returned largely variable results for this gene, likely due to individual batch effects. Alternatively, we cannot rule out that BV2 cells might require prolonged exposure to NO (>24 h) to effectively up-regulate *Itgam* gene expression, and such delayed response was not captured in our experimental setting.

In parallel with these studies, our investigations demonstrated that AZE treatment also triggered apoptotic cell death. Cell viability assays, flow cytometry and Western blotting all confirmed moderate cell loss at the highest concentrations of AZE. Annexin V/PI staining suggested mixed necrotic/apoptotic cell death; however, the increased expression of BAX protein levels and the gross microscopic observations of sparse cells with pyknotic nuclei points more towards apoptotic-like cell death. Additional observations to confirm whether AZE activates UPR-initiated cell death in BV2 microglia are warranted. Furthermore, if UPR is identified as the cause of AZE-mediated apoptosis, it would be interesting to determine whether this pathway leads the activation of the intrinsic or extrinsic apoptotic pathways, or both. In fact, there is evidence that ER-stress/UPR can also activate the extrinsic pathway via TRAIL receptor signalling [43] in addition to the intrinsic one [14].

AZE neurotoxicity, as well as its possible link with MS pathogenesis was initially hypothesized in 2008 by Rubenstein and coworkers in 2008 [6]. However, no direct links with other neurodegenerative conditions have been reported so far. Our findings showing that AZE triggers both apoptosis and inflammation support and extend the idea of a broader neuropathological mechanism underlying the toxic effects of this npAA. However, more mechanistic studies are warranted to ascertain how these pathogenic pathways are regulated and perhaps, can be reversed.

In conclusion, the present work provides novel evidence to indicate that AZE is both toxic and pro-inflammatory in BV2 microglia. The underlying mechanism still needs to be elucidated; however, published data supports a role for ER-stress and perhaps mitochondrial dysfunction as the two main pathogenic mechanisms. Understanding the detrimental activity of AZE and other npAAs is of great importance, as it may serve to raise awareness on the importance of monitoring consumption (or other means of exposure) to these potentially neurotoxic molecules.

## Figures and Tables

**Table 1:** Oligonucleotide primers sequences used to amplify the genes of interest. Sequences were optimised for use in real-time qPCR studies and SYBR green technology, with predicted amplicons < 165 bp.

Gene	Forward Sequence 5′–3′ Reverse Sequence 3′–5′	Tm (°C)	Product Size	Accession No.
*IL-1* *β*	GCTACCTGTGTCTTTCCCGT CATCTCGGAGCCTGTAGTGC	59.68 60.25	164	NM_008361.4
*Itgam*	GAGCAGGGGTCATTCGCTAC GCTGGCTTAGATGCGATGGT	60.53 60.53	94	NM_001082960.1
*Il-6*	CCCCAATTTCCAATGCTCTCC CGCACTAGGTTTGCCGAGTA	59.24 60.11	141	NM_031168.2
*Cd68*	CTCCCACCACAAATGGCACT CTTGGACCTTGGACTAGGCG	60.54 60.11	95	NM_001291058.1
*Mhc-2a*	CAAGCTGTCTTATCTCACCTTCA ATCTCAGGTTCCCAGTGTTTCA	60.34 61.81	108	NM_010378.3
*Mmp-9*	ATCATAGAGGAAGCCCATTACAG TTTGACGTCCAGAGAAGAAGAAA	59.86 59.96	129	NM_013599.4
*Nos2*	TACCAAAGTGACCTGAAAGAGG TCATCTTGTATTGTTGGGCTGA	60.06 59.96	89	NM_010927.4
*Il-10*	GCATGGCCCAGAAATCAAGG GAGAAATCGATGACAGCGCC	59.54 59.42	91	NM_010548.2
*Arg-1*	ACAAGACAGGGCTCCTTTCAG TTAAAGCCACTGCCGTGTTC	59.93 59.05	105	NM_007482.3
*Aif1*	ACGTTCAGCTACTCTGACTTTC GTTGGCCTCTTGTGTTCTTTG	60.23 60.18	107	NM_001361501.1
*Bdnf*	CGAGTGGGTCACAGCGGCAG GCCCCTGCAGCCTTCCTTGG	60.04 59.97	160	NM_007540.4
*S18*	CCCTGAGAAGTTCCAGCACA GGTGAGGTCGATGTCTGCTT	59.60 59.75	145	NM_011296.2

**Table 2:** Antibodies used in Western Blots.

Antibody	Source	Predicted Band Size	Dilution
Bcl2	ab182858, Abcam	26 kDa	1:2000
BAX	ab32503, Abcam	21 kDa	1:1000
Arg1	GTX109242, GeneTex	35 kDa	1:1000
BDNF	GTX132621, GeneTex	28 kDa	1:1000 (WB) 1:500 (IHC)
Iba1	GTX100042, GeneTex	17 kDa	1:500 (WB) 1:250 (IHC)
iNOS	GTX60599, GeneTex	32 kDa	1:1000
IL-6	GTX110527, GeneTex	24 kDa	1:1000 (WB)
GAPDH	VPA00187, Bio-Rad	37 kDa	1:2000
Goat anti Rabbit IgG HRP (Secondary)	STAR208P, Bio-Rad		1:10,000

WB = Western blot. IHC = Immunohistochemistry.

## Data Availability

All data generated or analysed during this study are included in this published article.

## Supplementary Materials

The following supporting information can be downloaded at: https://www.mdpi.com/article/10.3390/cimb44100308/s1, Figure S1: Gating of Annexin V-FITC/PI staining in flow cytometry.
