# Supplementary figures and images for: Pro-Inflammatory and Pro-Apoptotic Effects of the Non-Protein Amino Acid L-Azetidine-2-Carboxylic Acid in BV2 Microglial Cells
Source: Curr Issues Mol Biol. 2022 Sep 28;44(10):4500–16. doi: 10.3390/cimb44100308 (PMC9600089; doi:10.3390/cimb44100308)

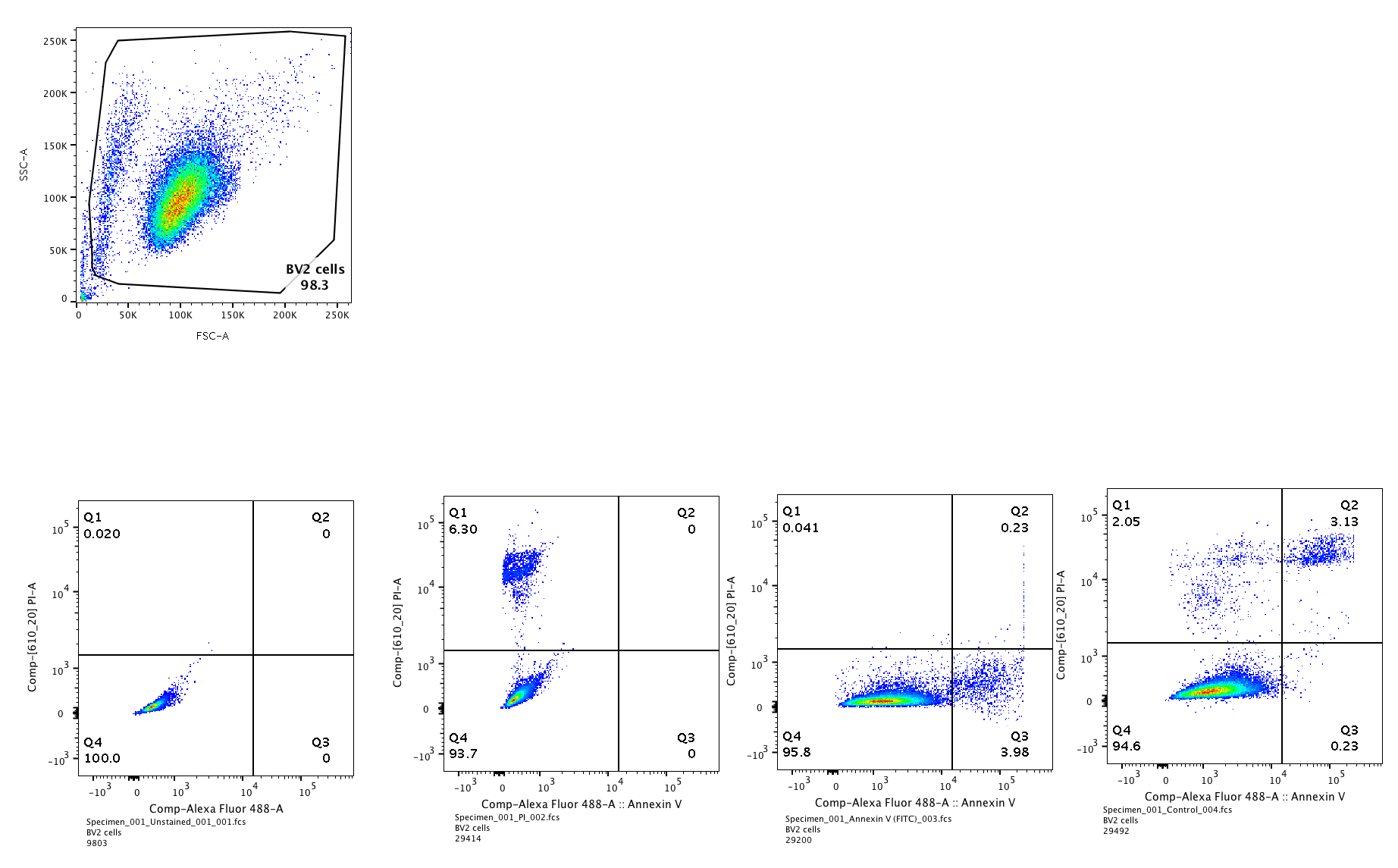

Supplement: Supplementary file 1 [file cimb-44-00308-s001.zip › cimb-1888102-supplementary.tiff]
